# Putative periodontopathic bacteria and herpes viruses interactions in the subgingival plaque of patients with aggressive periodontitis and healthy controls

**DOI:** 10.1002/cre2.80

**Published:** 2017-10-27

**Authors:** Amal Elamin, Raouf Wahab Ali, Vidar Bakken

**Affiliations:** ^1^ Department of Health Sciences, College of Natural and Health Sciences Zayed University Dubai UAE; ^2^ Department of Clinical Science University of Bergen Bergen Norway; ^3^ Department of Periodontology University of Science and Technology Omdurman Sudan

**Keywords:** aggressive periodontitis, cytomegalovirus, Epstein–Barr virus, loop‐mediated isothermal amplification (LAMP), periodontal microbiota, subgingival plaque

## Abstract

The microbial profile of aggressive periodontitis patients is considered to be complex with variations among populations in different geographical areas. The aim of this study was to assess the presences of 4 putative periodontopathic bacteria (Aggregatibacter actinomycetemcomitans, Porphyromonas gingivalis, Tannerella forsythia, and Treponema denticola) and 2 periodontal herpes viruses (Epstein–Barr virus type 1 [EBV‐1] and human cytomegalovirus [HMCV]) in subgingival plaque of Sudanese subjects with aggressive periodontitis and healthy controls. The study group consisted of 34 subjects, 17 aggressive periodontitis patients and 17 periodontally healthy controls (14–19 years of age). Pooled subgingival plaque samples were collected and analyzed for detection of bacteria and viruses using loop‐mediated isothermal amplification. Prevalence of subgingival A. actinomycetemcomitans, HCMV, and P. gingivalis were significantly higher among aggressive periodontitis patients than periodontally healthy controls. Coinfection with A. actinomycetemcomitans, HCMV, and/or EBV‐1 was restricted to the cases. Increased risk of aggressive periodontitis was the highest when A. actinomycetemcomitans was detected together with EBV‐1 (OD 49.0, 95% CI [2.5, 948.7], p = .01) and HCMV (OD 39.1, 95% CI [2.0, 754.6], p = .02). In Sudanese patients, A. actinomycetemcomitans and HCMV were the most associated test pathogens with aggressive periodontitis.

## 1 INTRODUCTION

There is ample evidence that the etiopathogenesis of aggressive periodontitis is multifactorial, involving genetic predisposition (de Carvalho, Tinoco, Govil, Marazita, & Vieira, 2009) and complex interactions between specific oral microbiota and the host tissues (Socransky & Haffajee, 2005). Other risk factors also play a role in the initiation and progression of attachment loss in aggressive periodontitis (Albandar & Rams, 2002; Contreras et al., 2015; Stabholz, Soskolne, & Shapira, 2010).

Identification of the microbial composition of infectious diseases is an important step for establishing clinical diagnosis and treatment planning. However, in the case of aggressive periodontitis, the oral biofilms constitute complex and dynamic bacterial mixed‐species communities, and this hampers the task of accurately identifying the causative periodontopathic microorganisms (Haffajee & Socransky, 2006).

Gram‐negative anaerobic bacteria residing in the dental pocket have extensively been incriminated as the primary etiological factors for initiation and progression of aggressive periodontitis (Armitage, 2010). Among these bacteria, *Aggregatibacter actinomycetemcomitans* has been implicated as a key putative pathogen in aggressive periodontitis (Zambon, 1985). Other studies have shown higher prevalence of other bacteria associated with periodontal destruction, namely, *Porphyromonas gingivalis*, *Tannerella forsythia*, *Campylobacter rectus*, *Prevotella intermedia*, and *Tannerella denticola* (Armitage, 2010; Faveri et al., 2009; Gajardo et al., 2005; van Winkelhoff, Loos, van der Reijden, & van der Velden, 2002). However, periodontal pathogens may exist in subgingival sites without causing periodontal attachment loss (Aberg et al., 2009), suggesting a host response role and/or differences with respect to the pathogenic potential of these bacteria (Kumar et al., 2006; Papapanou, 2002).

It has been suggested that herpes viruses, particularly human cytomegalovirus (HCMV) and Epstein–Barr virus type 1 (EBV‐1), could induce periodontal destruction (Rodrigues, Teixeira, Kustner, & Medeiros, 2015; Slots, 2005; Slots, 2015). A proposed viral–bacterial paradigm (Slots, 2010a) is supported by the notion that localized aggressive periodontitis lesions may be associated with high genome‐copy counts of herpes viruses, suggesting their involvement in the course of the disease (Saygun, Kubar, Ozdemir, Yapar, & Slots, 2004; Slots, 2010b; Ting, Contreras, & Slots, 2000). Insofar, the ability of *A. actinomycetemcomitans* and *P. gingivalis* to invade epithelial cells, which are the primary site for infection of HCMV, is consistent with the proposed model (Eick & Pfister, 2004; Saygun et al., 2004; Teughels, Sliepen, Quirynen, et al., 2007).

We have previously conducted a cross‐sectional study among 1,200 high school students, aged 13–19 years, in Khartoum State, Sudan, and found a high prevalence of aggressive periodontitis (3.4%; Elamin, Skaug, Ali, Bakken, & Albandar, 2010). In a case–control study in the same population, we examined 17 aggressive periodontitis cases and 17 controls and detected *A. actinomycetemcomitans* in the subgingival plaque of 12 cases (70.6%) and only one control (5.9%). This suggested a strong association between aggressive periodontitis and subgingival infection with *A. actinomycetemcomitans* in this population (odds ratio 38.4; Elamin, Albandar, Poulsen, Ali, & Bakken, 2011).

Comparison of the periodontal microbial profile of aggressive periodontitis cases and periodontally healthy controls may provide an important insight into the microbiological etiology of the disease.

The aim of the present study was to assess the presence of selected herpes viruses EBV‐1 and HCMV and periodontopathic bacteria *A. actinomycetemcomitans*, *P. gingivalis*, *T. forsythia*, and *T. denticola* in the subgingival plaque of aggressive periodontitis cases and controls.

## 2 MATERIALS AND METHODS

### 2.1 Subject population

Thirty‐four subjects participated in the present study: 17 subjects with localized aggressive periodontitis (mean age 15.5, ±1.6 years) and 17 subjects (mean age 15.6, ±1.5 years) with no clinical periodontal attachment loss (controls). The aggressive periodontitis patients (cases) and periodontally healthy subjects (controls) were identified in a large survey of aggressive periodontitis, which was conducted among high school students in Khartoum State, Sudan. One thousand two hundred (1,200) students participated in the survey, and these were selected using a multistage, stratified sampling design from a target population of approximately 150,000 students and 744 high schools. Forty‐one patients with localized aggressive periodontitis were identified in the survey, a disease prevalence rate of 3.4%. Of these, 17 subjects (cases) agreed to participate in the present study, a response rate of 41.5%. An interview with the subjects showed that the most common reasons for nonresponse were cultural belief that the study could lead to transmission of infectious diseases and dental fear. A detailed description of the study design and the inclusion criteria are published elsewhere (Elamin et al., 2010).

Aggressive periodontitis patients were defined as subjects with rapid periodontal attachment loss on multiple teeth, but otherwise systemically healthy (Albandar & Rams, 2002). In the field survey, young subjects with at least four teeth with interproximal sites showing ≥4 mm attachment loss or at least three teeth with interproximal sites showing ≥5 mm attachment loss were classified in the aggressive periodontitis group (Elamin et al., 2010; Susin & Albandar, 2005).

The periodontally healthy controls were defined as subjects who showed no clinical attachment loss or clinical signs of gingivitis in any examined tooth, and they were matched to cases on gender and ethnicity.

In the survey, students received periodontal examination at their schools by one examiner (A. Elamin). After the conclusion of the survey, controls were invited to participate in the present study. The collection of periodontal samples was performed at the home of each participant. Aims and objectives of the study were explained to the subjects, and only consenting subjects were included in the study. Subjects were excluded from the study if they had a contagious systemic infection and had used systemic antibiotics or antiviral treatment or received periodontal treatment within 3 months prior to the study.

### 2.2 Ethical consideration

The protocol was approved by ethical committees at the following institutions in Sudan: University of Science and Technology, Omdurman; Ministry of Health, and Ministry of Education. A written consent was obtained from each of the participants and parents or guardians as well as from school principals. Study participants were informed of the study objectives and methods and that their participation is voluntary and they may withdraw at any time.

### 2.3 Subgingival plaque samples

Pooled subgingival plaque samples were collected from 34 subjects, consisting of 17 cases and 17 controls. Prior to sampling, the sites were isolated with sterile cotton rolls, and supragingival deposits were gently removed using sterile periodontal curettes. Four subgingival plaque samples per subject were collected using paper points (#35, Zipperer, Munich, Germany) from the deepest periodontal pocket, one sample from each quadrant. At each site, two sterile paper points were inserted to the bottom of the periodontal pocket and kept for 10 s; then paper points from each subject were pooled immediately into a 2‐ml vial containing VMGA III transport medium (Dahlen, Pipattanagovit, Rosling, & Moller, 1993). Samples were transported within 24 hr after collection to the laboratory at the Department of Clinical Science, University of Bergen, Norway, and were stored at −80 °C until further analyses, which was performed within 18 months from the conclusion of periodontal sampling procedure.

### 2.4 Bacterial strains and culture conditions

The following strains were used as positive controls in this study: *A. actinomycetemcomitans* Y4 (ATCC 43718), *P. gingivalis* FDC 381, *T. forsythia* FDC 338, and *T. denticola* ATCC 35405, B95‐8. Double distilled water and *Fusobacterium nucleatum* ATCC 25586 were used as negative controls.

*A. actinomycetemcomitans* and *P. gingivalis* strains were cultivated on fastidious anaerobic blood agar plates (Lab M, Bury, UK), which were incubated anaerobically (5% CO_2_, 10% H_2_, and 85% N_2_) using Anoxomat system (MART Microbiology BV, Lichtenvoorde, The Netherlands) at 37 °C for 4 to 5 days.

*T. forsythia* was cultivated on tryptic soy agar with n‐acetylmuramic acid (TSA‐NAM) anaerobically at 37 °C for 3 to 4 days (Wyss, 1989). The viral DNA (positive controls) for EBV‐1 and HCMV were provided by the laboratory of the Section of Microbiology, Haukeland Hospital, Bergen, Norway. Cultured *T. denticola* was kindly provided by Dr. Ulf R. Dahle, Norwegian Institute of Public Health, Oslo, Norway.

### 2.5 Sample preparation and DNA extraction

The subgingival plaque samples were thawed at room temperature; paper points were immersed in tubes containing 180 μl of buffer T1 from the NucleoSpin Tissue kit (Macherey‐Nagel, Duren, Germany) and were then mixed vigorously for 60 s. DNA template from the subgingival plaque was obtained using the NucleoSpin Tissue kit according to the manufacturer's protocol. DNA from the reference strains was purified using E.Z.N.A. Bacterial DNA kit (Omega Bio‐Tek, Norcross, GA, USA). Template from *T. denticola* was prepared as follows: 30 μl of *T. denticola* bacterial cells were suspended in 100 μl of phosphate buffer saline. The suspension was boiled for 5 min, and 5.5 μl was used as a template.

### 2.6 Loop‐mediated isothermal amplification

In this study, we used 16S rRNA‐based loop‐mediated isothermal amplification (LAMP) for detection of periodontopathic bacteria as described previously (Miyagawa et al., 2008). Moreover, LAMP method was used to amplify viral DNA of EBV‐1 and HCMV as described previously (Iwata et al., 2006; Suzuki et al., 2006). Primers used in this study are listed in Table 1. LAMP was performed in a total reaction mixture volume of 25 μl of the following: 1.6 μM of each forward inner primer and backward inner primer, 0.2 μM of the outer primers F3 and B3, 0.4 μM of the loop primers LF and LB, 1.4 mM each of the four deoxynucleoside triphosphates, 8 U of the *Bst* DNA polymerase large fragment (New England Biolabs), 0.8 M betaine (Sigma), 20 mM Tris‐HCl (pH 8.8), 10 mM KCl, 10 mM (NH_4_)_2_SO_4_, 8 mM MgSO_4_, 0.1% Tween 20, and template DNA in a volume of up to 5.5 μl. The mixtures were incubated at 63, 64, and 65 °C using a thermocycler for 60 min and then heated at 80 °C for 2 min to terminate the reaction. LAMP amplicon was detected by visual inspection using a turbidity derived from the white precipitate of magnesium pyrophosphate and by 3% agarose gel electrophoresis and followed by staining with ethidium bromide.

**Table 1:** Oligonucleotide primers for loop‐mediated isothermal amplification

Target species	Primers	Sequences
*Aggregatibacter actinomycetemcomitans* c	FIP	5′‐CCCCACGCTTTCGCACATCATACCGAAGGCGAAGGCAG‐3′
Y4 (ATCC 43718)^b^	BIP	5′‐AGATACCCTGGTAGTCCACGCTTTCGGGCACCAGGGCTAAAC‐3′
	F3	5′‐TGCGTAGAGATGTGGAGGAA‐3′
	B3	5′‐GGCGGTCGATTTATCACGT‐3′
	LB	5′‐AAACGGTGTCGATTTGGGGAT‐3′
*Porphyromonas gingivalis* c	FIP	5′‐CACCACGAATTCCGCCTGCCTGAGCGCTCAACGTTCAGCC‐3′
FDC 381	BIP	5′‐ATCACGAGGAACTCCGATTGCGCGCCTTCGTGCTTCAGTG‐3′
	F3	5′‐GGTAAGTCAGCGGTGAAACC‐3′
	B3	5′‐GCGTGGACTACCAGGGTAT‐3′
	LB	5′‐GCAGCTTGCCATACTGCGA‐3′
*Tannerella denticola* c	FIP	5′‐CATCCTGAAGCGGAGCCGTAGTACCGAATGTGCTCATTTAC‐3′
ATCC 35405	BIP	5′‐GCTGGTTGGTGAGGTAAAGGCCATCTCAGTCCCAATGTGTCC‐3′
	F3	5′‐CCCTGAAGATGGGGATAGCT‐3′
	B3	5′‐TGCCTCCCGTAGGAGTTT‐3′
	LB	5′‐CACCAAGGCAACGATGGGTAT‐3′
*Tannerella forsythia* c	FIP	5′‐CCATCCGCAACCAATAAATCTCTAATACCTCATAAAACAGG‐3′
FDC 338^a^	BIP	5′‐TAAGCCATCGATGGTTAGGGCGTGTCTCAGTACCAGTGTG‐3′
	F3	5′‐GATAACCCGTAGGAGTTTG‐3′
	B3	5′‐TGCCTCCCGTAGGAGTCT‐3′
	LB	5′‐GTTCTGAGAGGAAGGTCCCC‐3′
Epstein–Barr virus^d^	FIP	5′‐CTAGCAACGCGAACCCCCTT‐GGAGTGGGCTTGTTTGTGAC
	BIP	5′‐CTCAGTCCAGCGCGTTTACGT‐CTTTATACCAGGGGCAGTGG
	F3	5′‐AGAGGAATAAGCCCCCAGAC‐3′
	B3	5′‐ACCAGAAATAGCTGCAGGAC‐3′
	LF	5′‐GGGCCCTGACCTTTGGTGAA‐3′
	LB	5′‐GCAGCCAATTGTCAGTTCTAGG‐3′
Human cytomegalovirus^e^	FIP	5′‐CGTTGGCGTAGCCATTGGGGAGGGGTTTTTGAGGAAGGTG‐3′
	BIP	5′‐CGCTCATGAGGTCGTCCAGACGTGGAGGACAAGGTAGTCGA‐3′
	F3	5′‐CACGAGGATGATGGTGAAGG‐3′
	B3	5′‐AACTCGTACAAGCAGCGG‐3′
	LF	5′‐AACGCCTTCGACCACGGAGG‐3′
	LB	5′‐TTGAGGTAGGGCGGTAGCGGG‐3′

a FDC is the collection of Forsyth Dental Centre.

b ATCC is the American Type Culture Collection.

c 16S rRNA‐based primers as described by (Miyagawa et al., 2008).

d Epstein–Barr virus detection as described by (Iwata et al., 2006).

e Human cytomegalovirus detection as described by (Suzuki et al., 2006).

### 2.7 Data analysis

The significance of differences of detection frequencies of periodontopathic microorganisms between the two subject groups was analyzed using Mantel–Haenszel chi‐square test and Fisher's exact test. Student's *t* test was used for comparison of means.

The odds ratio and the 95% confidence interval were calculated, and association between pathogens and the disease were examined using two‐by‐two contingency tables. Wald test was used to test significance. The statistical analysis was performed using the software STATA (v11.0, Stata Corporation, College Station, Texas, USA).

## 3 RESULTS

The demographic characteristics and clinical parameters of the studied population are presented in Table 2. The cases and controls were balanced with regard to gender and ethnicity distribution.

**Table 2:** Demographic characteristics and clinical parameters of aggressive periodontitis cases and healthy controls

	Aggressive periodontitis *n =* 17	Periodontally healthy *n* = 17	Total	*p* value
Sex				.7
Female	9 (47.4%)	10 (52.6%)	19	
Male	8 (53.3%)	7 (46.7%)	15	
Ethnicity^a^				.7
African	4 (50.0%)	4 (50.0%)	8	
Afro‐Arab	12 (48.0%)	13 (52.0%)	25	
Age in years‐mean	15.4 (1.6)	15.6 (1.5)		.6
PPD (mean ± *SD*) mm	6.9 ± 1.5	1.4 ± 0.5	NA	.01
CAL	4.8 ± 1.2	Nil	NA	.001
BOP (%) of positive sites	62.4	Nil	NA	.001
Number of teeth present (mean ± *SD*)	27.6 ± 4.6	28.3 ± 2.3	NA	.3

*Note*. *SD* = standard deviation; PPD = probing pocket depth; CAL = clinical attachment loss; BOP = bleeding on probing; NA = not applicable.

a One subject was excluded from the table because the ethnicity was unknown.

**Figure 1:**
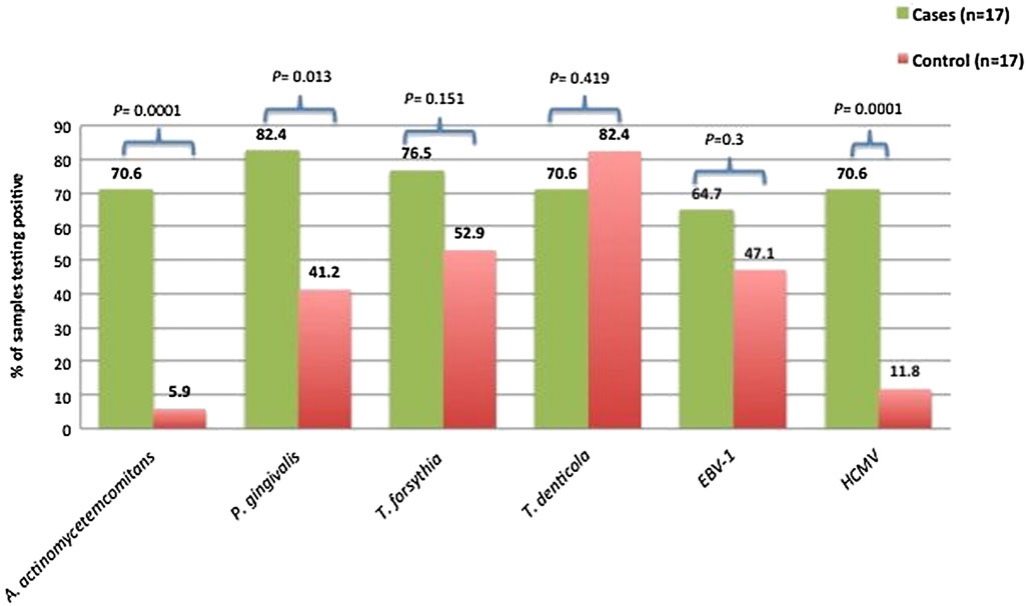
The percentage of samples testing positive for targeted periodontal pathogens and viruses in the pooled subgingival plaque of the cases and controls. EBV‐1 = Epstein–Barr virus type 1; HCMV = human cytomegalovirus; *A. actinomycetemcomitans* = *Aggregatibacter actinomycetemcomitans*; *P. gingivalis* = *Porphyromonas gingivalis*; *T. forsythia* = *Tannerella forsythia*; *T. denticola* = *Tannerella denticola*

We used LAMP method for the detection of six microorganisms:

*A. actinomycetemcomitans*, *P. gingivalis*, *T. forsythia*, *T. denticola*, EBV‐1, and HCMV in the subgingival plaque of cases and controls. *A. actinomycetemcomitans*, *P. gingivalis*, *T. forsythia*, and *T. denticola* were detected in 70–82% and 5–82% of the subgingival plague of cases and controls, respectively. Subgingival EBV‐1 and HCMV occurred in 64–70% and 11–47% of cases and controls, respectively (Figure 1).

**Figure 2:**
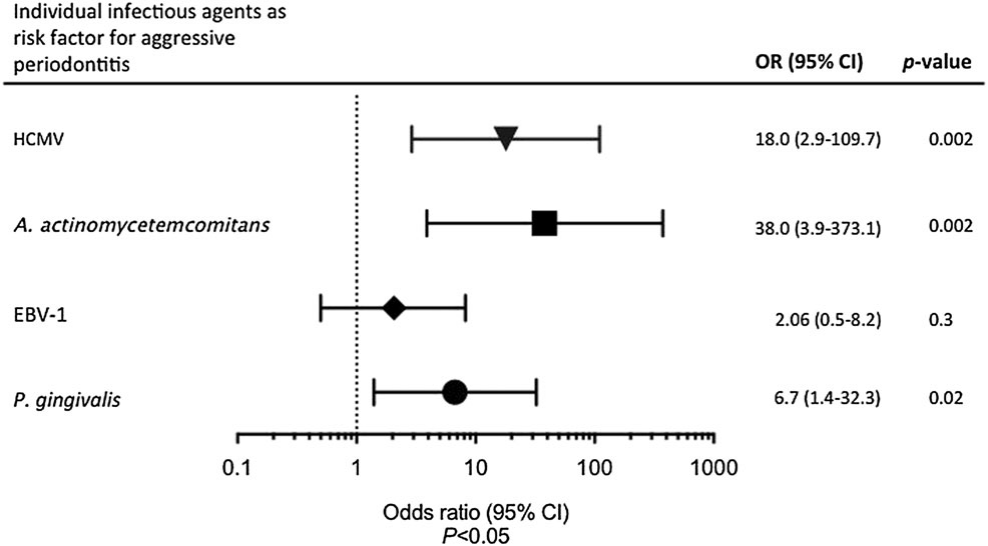
Forest plot of the association between individual periodontal pathogens and viruses and the risk of aggressive periodontitis. EBV‐1 = Epstein–Barr virus type 1; HCMV = human cytomegalovirus; OR = odds ratio; *A. actinomycetemcomitans* = *Aggregatibacter actinomycetemcomitans*; *P. gingivalis* = *Porphyromonas gingivalis*

Significant associations (*p* < .05) were found between *A. actinomycetemcomitans*, HCMV, and *P. gingivalis* and aggressive periodontitis (Figure 2). However, no significant association was found between EBV‐1 and the disease.

Coinfection with *P. gingivalis* and EBV‐1 was significantly higher among cases than controls (*p* < .001). Also, coinfection with *P. gingivalis* and HCMV was found to be significantly higher among cases than controls (*p* < .001). Eight cases were found to harbor combinations of *P. gingivalis*, EBV‐1, and HCMV revealing a significantly higher detection in this studied group compared to controls (*p* < .001). Aggregates of *A. actinomycetemcomitans*, EBV‐1, and/or HCMV were confined to cases (*p* = .0001).

The odds ratio of having aggressive periodontitis was substantially higher when *A. actinomycetemcomitans* was detected together with EBV‐1 (odds ratio 49.0, 95% CI [2.5, 948.7], *p* = .01) or HCMV (odds ratio 39.1, 95% CI [2.0, 754.6], *p* = .02). Moreover, presence of the two tested herpes viruses with *A. actinomycetemcomitans* or *P. gingivalis* increased the risk of having aggressive periodontitis by 31.3 folds (odds ratio 31.3, 95% CI [1.6, 604.1], *p* = .02). *P. gingivalis* and EBV‐1 increased the risk of having aggressive periodontitis by 10.7 folds (odds ratio 10.7, 95% CI [1.8, 62.5], *p* = .008; Table 3).

**Table 3:** Associations between combination of infectious agents and disease condition

Aggressive periodontitis	Odds ratio [95% CI]	*p* value*
*A. actinomycetemcomitans* and EBV‐1	49.0 [2.5, 948.7]	.01
*A. actinomycetemcomitans* and HCMV	39.1 [2.0, 754.6]	.02
*A. actinomycetemcomitans*, EBV‐1, and HCMV	31.3 [1.6, 604.1]	.02
*P. gingivalis* and EBV‐1	10.7 [1.8, 62.5]	.008
*P. gingivalis* and HCMV	29.3 [3.1. 278.8]	.003
*P. gingivalis*, EBV‐1, and HCMV	31.3 [1.6, 604.1]	.02

*Note*. EBV‐1 = Epstein–Barr virus type 1; HCMV = human cytomegalovirus; *A. actinomycetemcomitans = Aggregatibacter actinomycetemcomitans*; *P. gingivalis* = *Porphyromonas gingivalis*.

* *p* < .05.

## 4 DISCUSSION

We used the LAMP method for detection of four putative periodontopathic bacteria and two periodontal herpes viruses in pooled subgingival plaque of localized aggressive periodontitis cases and controls. In this population, there was strong significant association between aggressive periodontitis and subgingival infection with *A. actinomycetemcomitans*, where *A. actinomycetemcomitans* was significantly higher among cases than in controls (*p* = .0001), and it increased the risk of aggressive periodontitis by 38.4 (Elamin et al., 2011). Conversely, *P. gingivalis* was found to be the most prevalent subgingival target bacteria detected among cases (82.4%), with frequency of detection significantly higher among cases than in controls (*p* = .013). *P. gingivalis* was detected in 41.2% of the controls, whereas *A. actinomycetemcomitans* was detected in only 5.9% of the controls. These results suggest a superior role for *A. actinomycetemcomitans* and to lesser extent *P. gingivalis* in pathogenesis of aggressive periodontitis. The observation that frequency of detection of *P. gingivalis* is higher among controls than that of *A. actinomycetemcomitans* suggests that *P. gingivalis* constitutes a portion of the subgingival normal flora of these subjects in this age group and may play a dual role as normal flora and pathogen, that is, a commensal opportunistic pathogen (Armitage, 2010). Moreover, the presence of *P. gingivalis* among the control group could be prognostic for future chronic periodontal disease (Armitage, 2010). Further longitudinal studies will be needed to test these possibilities. On the other hand, individuals may harbor putative pathogens for a long time without developing periodontal diseases, suggesting wide variations with regard to the virulence of the pathogens and differences in the hosts' responses (Armitage, 2010; Kilian, Frandsen, Haubek, & Poulsen, 2006). However, nonquantitative data provide limited information, and conclusions drawn from these results must be made with caution. Furthermore, HCMV was found to be significantly higher among cases than controls (70.6%, 11.8%, *p* = .000) and was accountable for increased risk of the disease by 18 folds. These findings are in agreement with reports on the proposed role of HCMV and other herpes viruses on triggering periodontal destruction (Contreras et al., 2015; Slots, 2011; Slots & Contreras, 2000). It is suggested that the presence of herpes viruses in periodontal lesions may mediate the release of cytokines and chemokines from host inflammatory cells and interferes with antibacterial immune mechanisms, providing a suitable setting for further colonization by periodontopathic bacteria, elevated virulence of the resident pathogens, and subsequently more periodontal breakdown (Slots, 2010a). Other studies have reported significant association between activation of HCMV infection in periodontally disease‐active sites and advancing aggressive periodontitis, suggesting the involvement of the virus in the course of the disease (Ting et al., 2000).

In the present study, we investigated periodontopathic bacterial–viral synergy in the subgingival plaque of Sudanese subjects. Our results show evident association between aggressive periodontitis and subgingival harboring of *A. actinomycetemcomitans*, EBV‐1, and HCMV. Markedly, subgingival coinfection with *A. actinomycetemcomitans*, EBV‐1, and/or HCMV was confined to the cases. None of the controls harbored the combinations of *A. actinomycetemcomitans*, EBV‐1, and/or HCMV. We found that dual infection with *P. gingivalis* and HCMV was the most prevalent combination of the target microorganisms detected among the cases (64.7%). Likewise, the frequency of detection of *P. gingivalis–*HCMV combination was significantly higher among cases than controls (64.7%, 5.9%, *p* = .001). The odds ratio of having aggressive periodontitis increased multiplicatively in subjects with dual infection with HCMV and *P*. *gingivalis* (odds ratio 29.3). Correspondingly, in a case–control study, Michalowicz, Ronderos, Camara‐Silva, Contreras, & Slots, (2000) investigated the presence of periodontopathic bacteria and periodontal herpes viruses in the subgingival plaque of 100 adolescents in Jamaica. The authors found that the association with aggressive periodontitis and the extent of attachment loss was the strongest when there was dual infection with *P. gingivalis* and HCMV (odds ratio of 3.9; Michalowicz et al. (2000). Similarly, Kamma et al. detected the presence of HCMV, EBV‐1, and periodontopathic bacteria in disease‐active and disease‐stable sites of 16 subjects with aggressive periodontitis in Greece (Kamma et al., 2001). The authors reported that periodontal active sites were strongly associated with coinfection with HCMV and EBV‐1. Moreover, a close association was found between the presence of HCMV and *P. gingivalis* (Slots, Kamma, & Sugar, 2003). However, variations in detection of periodontal herpes viruses among studies may be attributed to differences in the periodontal status of the studied subjects and diversity of the methods used to detect the viruses or due to true ethnic/geographical disparities in the occurrence of the periodontal herpes viruses (Slots, 2010a).

Our findings among cases and controls demonstrated significant association between the presence of test herpes viruses and the infection with *A. actinomycetemcomitans*. Principally, combinations of *A. actinomycetemcomitans*, HCMV, and/or EBV‐1 were restricted to the cases (*p* = .0001). The confinement of *A. actinomycetemcomitans*–herpes viruses subgingival infection to the group of aggressive periodontitis cases may suggest an important role of these microorganisms in the pathogenesis of the disease. Moreover, the highest risk of aggressive periodontitis was observed when subjects in the group studied were colonized with *A. actinomycetemcomitans* and any of the tested herpes viruses. In vitro studies suggested that HCMV may increase the adherence of *A. actinomycetemcomitans* to epithelial cells (Teughels et al., 2007). Accordingly, aggressive periodontitis lesions with active HCMV infection were found to be associated with high proportions of *A. actinomycetemcomitans* (Ting et al., 2000). Our results are in agreement with those of Imbronito, Okuda, Maria de Freitas, Moreira Lotufo, and Nunes (2008) who investigated the presence of herpes viruses and putative pathogens among Brazilian subjects with generalized aggressive periodontitis, chronic periodontitis, and gingivitis and are periodontally healthy using nested PCR and 16S rRNA‐based PCR (Imbronito et al., 2008). The authors found that *A. actinomycetemcomitans* was the most prevalent detected pathogen among the aggressive periodontitis group. However, the authors reported no difference in the frequency of detection of HCMV among the four groups, which is inconsistent with our results. The inconsistency between our results and those of Imbronito et al. (2008) may be due to the difference in the form of aggressive periodontitis tested, that is, localized aggressive periodontitis in the current study and generalized in the later study. It is well recognized that the two forms of aggressive periodontitis, localized and generalized, are considered two distinct entities with regard to their etiology and pathogenesis (Lang et al., 1999). Conversely, Michalowicz et al. (2000) found significant association between aggressive periodontitis, *P. gingivalis*, *A. actinomycetemcomitans*, and HCMV (*p* < .05; Michalowicz et al., 2000). In the present study, the finding that *A. actinomycetemcomitans* contributed to higher risk of aggressive periodontitis (odds ratio 38.4) than the combinations of the trio *A. actinomycetemcomitans*, HCMV, and EBV‐1 (odds ratio 31.3) may be attributed to variations in frequency of detection of the target pathogens (70.6% and 47.1%, respectively). Moreover, the variations maybe due to the age range of the participants. Some studies report that periodontal EBV‐1 and HCMV are more frequently detected in the older age group (>30 years; Bilichodmath et al., 2009), although age reports about herpes viruses periodontal colonization are controversial (Slots, 2015). Despite the circumstantial evidence of the etiologic role and/or coetiologic role of herpes viruses in the present study, more studies are needed to better understand the role of the viruses in periodontal disease.

It could be argued that the number of participants in the present study was relatively small. However, cases and controls were matched by gender and ethnicity and belonged to the same age group (13–19 years). Lists of eligible matched controls were prepared for each case, and then a control was randomly selected, which reinforced the study design. Every effort was made to explain to the participants about the sterilization method, the sampling procedures, study objectives, and the importance of the early diagnosis of the disease.

We assessed the presence of selected herpes viruses EBV‐1 and HCMV and periodontopathic bacteria *A. actinomycetemcomitans*, *P. gingivalis*, *T. forsythia*, and *T. denticola* in the subgingival plaque of aggressive periodontitis cases and periodontally healthy controls. In summary, *A. actinomycetemcomitans*, HCMV, and *P. gingivalis* were significantly higher among cases than controls, implicating these pathogens as risk factors for aggressive periodontitis in this population.

The highest risks of aggressive periodontitis were reported when *A. actinomycetemcomitans* was detected together EBV‐1 or HMC, respectively. Coinfection with *A. actinomycetemcomitans*, HCMV, and/or EBV‐1 was restricted to the cases. In conclusion, present findings in our study emphasize on the coinfection role of *A. actinomycetemcomitans*, *P. gingivalis*, EBV‐1, and HMC in aggressive periodontitis. However, these conclusions are based on a relatively small sample; thus, substantial increase in the sample size would help in formulating more definitive conclusions to questions raised about the etiology of aggressive periodontitis. Furthermore, more studies are needed in this population to compare the severity of aggressive periodontitis with the quantitative load of herpes viruses and the status of the viral infection (active vs. latent) using molecular approaches.

## CONFLICT OF INTEREST

The authors declare that they have no conflict of interests.
